# Good mid‐ to long‐term outcomes after meniscus bucket‐handle tear repair: A comparative analysis with and without anterior cruciate ligament reconstruction

**DOI:** 10.1002/jeo2.12093

**Published:** 2024-07-15

**Authors:** Johannes Pawelczyk, Ilias Fanourgiakis, Sven Feil, Maja Siebold, Ioannis Kougioumtzis, Rainer Siebold

**Affiliations:** ^1^ International Center for Orthopedics, ATOS Clinic Heidelberg Germany; ^2^ Ruprecht Karl University Heidelberg Germany; ^3^ Institute for Anatomy and Cell Biology Ruprecht Karl University Heidelberg Germany

**Keywords:** anterior cruciate ligament reconstruction, arthroscopic surgery, bucket‐handle meniscal tear, meniscus repair, osteoarthritis

## Abstract

**Purpose:**

To evaluate mid‐ to long‐term clinical outcomes after arthroscopic bucket‐handle meniscal tear (BHMT) repair and to assess the impact of concurrent anterior cruciate ligament reconstruction (ACLR).

**Methods:**

A comparative retrospective case series with blinded outcome assessment was conducted. All consecutive patients treated with arthroscopic BHMT repair with or without concurrent ACLR between 2001 and 2021 were eligible for inclusion. Fifty‐five patients with an average follow‐up of 7.3 ± 3.4 years were included in the analysis. Outcome measures comprised post‐operative IKDC Subjective Knee Form, Lysholm Score, Tegner Activity Scale, KOOS, and visual analogue scale (VAS) for satisfaction. Additionally, failure and reoperation rates were assessed.

**Results:**

The failure rate was 9%. Medial BHMT repair showed superior post‐operative IKDC scores compared to lateral meniscus repair (*p* = 0.038). Concurrent ACLR did not demonstrate any impact on post‐operative KOOS, IKDC, Tegner or patient satisfaction. The mean IKDC score at final follow‐up across both groups was 80.4 ± 17.8. The mean Lysholm score was 86.9 ± 16.7. Mean KOOS scores were (i) symptoms: 83.6 ± 18.3, (ii) pain: 90.2 ± 14.4, (iii) activities of daily living: 93.6 ± 15.1, (iv) sports: 78.3 ± 26.0 and (v) quality of life: 70.5 ± 24.5. Mean patient satisfaction (VAS) was 7.9 ± 2.5. The mean Tegner score was 4.9 ± 1.9. A consistent positive correlation between the number of sutures used and post‐operative outcome measures was observed but did not reach statistical significance for most items.

**Conclusion:**

Arthroscopic BHMT repair achieved good clinical outcomes and an acceptable failure rate of 9% at a mean follow‐up of 7 years, supporting the clinical value of meniscal repair, including large BHMTs. Concurrent ACLR showed no impact on clinical outcomes.

**Level of Evidence:**

Level IV (retrospective case series).

**Abbreviations**
ACLR: anterior cruciate ligament reconstruction
ADL: activities of daily living
BHMT: bucket‐handle meniscal tear
IKDC: International Knee Documentation Committee
KOOS: Knee Injury and Osteoarthritis Outcome Score
MRI: magnetic resonance imaging
n.s.: not significant
QoL: quality of life
SD: standard deviation
VAS: visual analogue scale

## INTRODUCTION

The menisci are known to play a crucial role in normal knee joint function, enhancing load distribution, shock absorption, joint stability, lubrication and congruence [4, 7, 34]. Meniscal tears are common injuries, with an estimated incidence of 222 per 100,000 in patients aged 18–55 [2]. Due to their unique morphology and often large size, bucket‐handle meniscal tears (BHMTs) comprise approximately 10% of meniscal lesions and often require surgery [26]. Historically, meniscectomy was routinely performed to treat BHMTs. More recent studies have demonstrated improved clinical outcomes and delayed onset of osteoarthritis with meniscal repair as opposed to meniscectomy [1, 12, 19, 23, 40], especially in younger patients, shifting the focus among knee surgeons towards meniscal preservation strategies [6], especially when facing large BHMTs, where resection leads to considerable loss of meniscal tissue.

Common approaches for addressing BHMTs include all‐inside, inside‐out and outside‐in repair [28]. Recent systematic reviews have reported an overall failure rate of 14.8%–29.3%, with BHMTs being significantly more likely to fail compared to other tear types [5, 8]. Concurrent anterior cruciate ligament reconstruction (ACLR) is speculated to improve BHMT repair outcomes [11, 22, 33, 37]. However, evidence in this regard remains conflicted [20, 36]. While several studies have explored meniscal repair techniques, few data exist on the mid‐ to long‐term outcomes of arthroscopic repair for large BHMTs. Thus, further research is warranted to determine the optimal treatment approach for large BHMTs.

The aim of this study was to evaluate the clinical outcomes after arthroscopic repair of large BHMTs involving the posterior horn and pars intermedia to assess the efficacy of this procedure in the mid‐ to long‐term and to examine the impact of concurrent ACLR. The primary hypothesis was that arthroscopic BHMT repair would achieve superior post‐operative patient‐reported outcome measures at final follow‐up with acceptable failure rates. Furthermore, it was hypothesised that concurrent ACLR would lead to superior post‐operative patient‐reported outcome measures compared to isolated BHMT repair.

## MATERIALS AND METHODS

### Study design and regulatory information

After obtaining institutional review board approval (ATOS Clinic Heidelberg, Ethics Commission, reference number: 32021), a comparative, single‐surgeon, retrospective case series with blinded outcome assessment was conducted at the International Center for Orthopedics, ATOS Clinic, Heidelberg, a tertiary referral institution for orthopaedic surgery and sports traumatology. The study aimed to (i) evaluate clinical outcomes after arthroscopic BHMT repair and to assess the influence of (ii) concurrent ACLR, (iii) medial versus lateral BHMT and (iv) the number of sutures used on patient‐reported outcome measures.

Written informed consent was obtained from all patients prior to enrolment. This study adhered to the Strengthening the Reporting of Observational Studies in Epidemiology guidelines for reporting observational research [39] and the Declaration of Helsinki [41]. Data supporting these findings are available from the corresponding author upon reasonable request.

### Patient selection, eligibility criteria and outcome assessment

**Figure 1:**
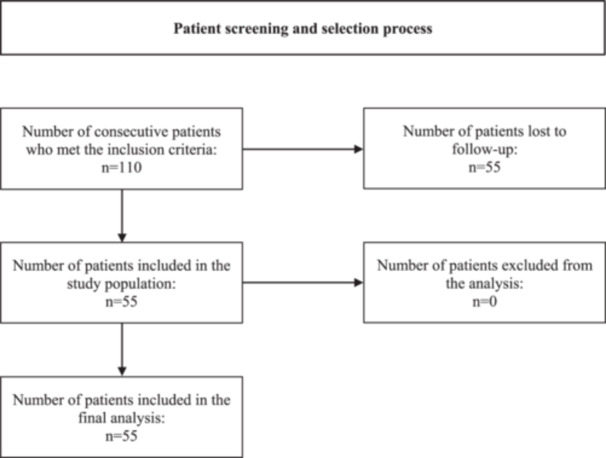
Patient screening and selection. Standard flow chart illustrating patient flow during the screening and selection process.

All consecutive patients treated with arthroscopic repair of large BHMTs between 2001 and 2021 were contacted for enrolment. Table 1 details eligibility criteria and Figure 1 summarises patient flow during the screening and selection process. The minimum follow‐up duration was 20 months.

**Table 1:** Eligibility criteria. Detailed inclusion and exclusion criteria. All patients received combined all‐inside and outside‐in repair.

Inclusion criteria	Exclusion criteria
Bucket‐handle meniscal tear	Chondromalacia (Outerbridge 3–4°)
Arthroscopic surgical repair using a hybrid technique (all‐inside and outside‐in sutures)	History of discoid meniscus
Surgery performed by the senior author between 2001 and 2021	History of complex traumatic injury between index surgery and final follow‐up, for example, high‐velocity trauma
Written informed consent	Rheumatoid or neurological disorder

Two independent raters (I.F. and S.F.), not involved in the surgical procedures and blinded to patient identity and type of surgery performed, assessed patients using internationally standardised and validated questionnaires. Data were collected according to a predetermined protocol, including the following patient‐reported outcome measures: International Knee Documentation Committee (IKDC) Subjective Knee Form [18], Knee Injury and Osteoarthritis Outcome Score (KOOS) [30], Tegner Activity Scale [35], Lysholm Score [25, 35], and visual analogue scale (VAS) [13] for patient satisfaction. Additionally, the failure rate was assessed, defining failure as conversion to arthroplasty or complete re‐rupture, confirmed in magnetic resonance imaging (MRI) or arthroscopy.

### Surgical technique

**Figure 2:**
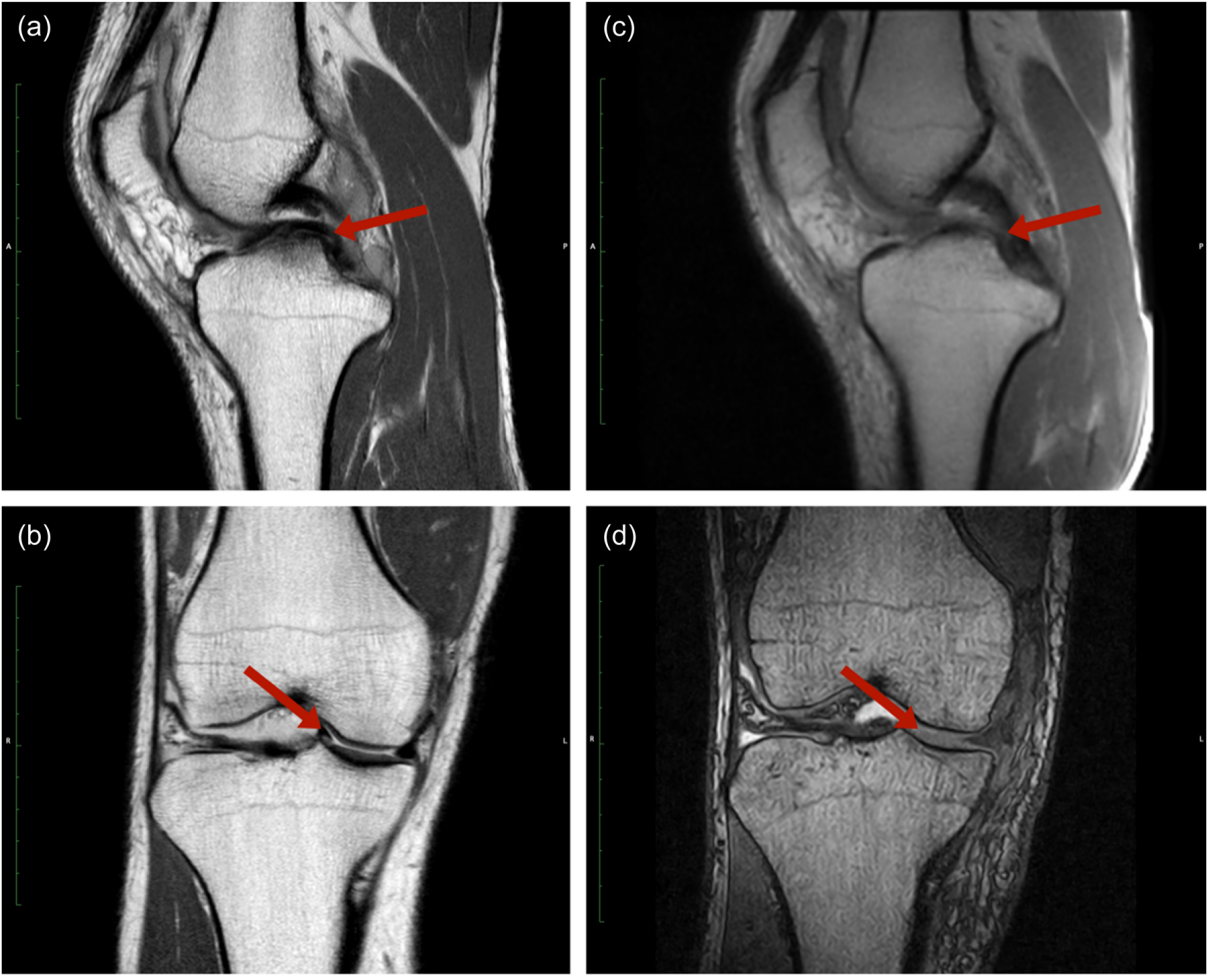
MRI findings. Illustrative MRI findings, depicting preoperative (a, b) and post‐operative (c, d) morphology. Arrows highlight relevant structures/pathologies. Preoperatively, visible dislocation of tear handle into intercondylar notch (a, b) with double PCL sign (a). MRI, magnetic resonance imaging; PCL, posterior cruciate ligament.

The senior author (R.S.) performed all surgeries using an arthroscopic approach. In brief, a comprehensive pre‐ and post‐operative examination was performed, including a complete physical examination of the affected extremity, radiographic evaluation, and MRI (Figure 2).

**Figure 3:**
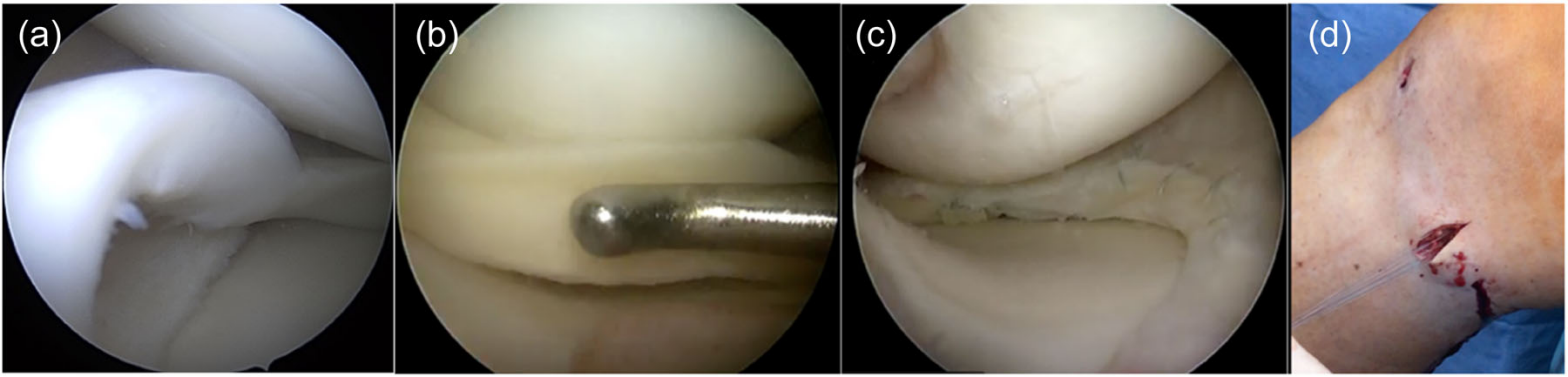
Surgical technique. Intraoperative view depicting the step of BHMT suturing: (a) visualisation of the lesion, (b) anatomical reduction of large, dislocated BHMT, (c) repaired meniscus with all‐inside and outside‐in sutures, and (d) minimal skin incision to tie knots onto the joint capsule. BHMT, bucket‐handle meniscal tear.

Upon initial arthroscopic visualisation, the BHMT was inspected, reduced, debrided, and finally sutured (Figure 3). All repairs were performed using a hybrid technique, combining all‐inside sutures (FAST‐FIX meniscal repair system, Smith & Nephew) in the posterior horn and outside‐in sutures in the pars intermedia of the medial or lateral meniscus. The all‐inside devices (an average of 3.9 in the posterior horn) and outside‐in sutures (an average of 7.2 in the pars intermedia) were always placed on both the upper and lower sides of the meniscus. If applicable, the concurrent ACLR was performed during the same procedure, using a double‐ or single‐bundle technique. ACLR was performed using hamstring grafts (gracilis and/or semitendinosus tendons), except for one case, where a quadriceps tendon graft was used. Femoral fixation was achieved using an EndoButton CL implant (Smith & Nephew). When performing a double‐bundle reconstruction, tibial fixation was achieved by tying a knot across the bone bridge between the two separate transtibial bone tunnels, without additional hardware. Otherwise, an interference screw was used. Bone marrow aspirate injection (*n* = 30) and notch microfracturing (*n* = 20) were used to improve healing response.

All patients followed the same post‐operative procedure. The affected extremity was placed in a hinged knee brace, with passive movement of the joint initiated on the first day post‐operatively. Flexion was limited to 60° in the first week and 90° in the second and third week. Free range of motion was permitted after three weeks. Only partial weight bearing was permitted for four weeks post‐operatively. Mild exercise was recommended after three months and return to sports at 6 or 12 months post‐operatively, depending on isolated meniscus repair or the combination with ACLR.

### Outcome measures

The primary outcome measure was the post‐operative IKDC score. Secondary outcome measures included post‐operative KOOS, Tegner, and Lysholm scores, as well as patient satisfaction (VAS) and failure rate. Subgroup analyses were performed to assess the influence of concurrent ACLR, number of sutures used, and medial versus lateral BHMT on post‐operative outcome measures, comparing (i) ACLR versus isolated BHMT repair, as well as (ii) medial versus lateral BHMT repair. The influence of the number of sutures used was assessed using the Tau B correlation coefficient (the number of sutures was considered the independent variable). Intra‐operative injection of bone marrow aspirate was considered as a possible confounder during the comparison of isolated BHMT repair versus BHMT repair with concurrent ACLR.

### Statistical analysis

Arithmetic mean, standard deviation (SD), and range were calculated for complete datasets (*n* = 55 cases with complete data for most study items). Groups were compared with Chi‐square (*χ* ^2^) tests and unpaired *t* tests. Non‐parametric Tau B tests were applied to determine whether the correlations between determinants and outcome parameters were significant. Due to the explorative design of this study, no alpha adjustment was performed. Complex analyses (e.g., multiple regression) were not applied, due to the small sample size. Data analysis was performed with IBM SPSS for Microsoft Windows (Version 27). All tests were two‐sided and a *p* value of <0.05 was considered significant.

## RESULTS

At a mean follow‐up of 7.3 ± 3.4 years, 55 patients were available for inclusion (34 male, 21 female), including 11 lateral and 46 medial BHMTs (two patients had concurrent medial and lateral tears). The average age at the time of index surgery was 30.3 ± 14.2 years. Tears were located in the red‐red zone in 94% of patients and 71% of bucket handles were dislocated into the intercondylar notch upon initial arthroscopic visualisation. The mean number of sutures used was 11 ± 3 (all‐inside devices plus outside‐in sutures). Patient and treatment demographic data are detailed in Table 2.

**Table 2:** Patient and defect characteristics. Detailed summary of patient and defect demographic data.

Characteristics	Coding/categories	*n* or range	Arithmetic mean ± SD or percentage
Sex	Male	34	61.8%
	Female	21	38.2%
Age at surgery	Years	12–63	30.3 ± 14.2
ACLR	Yes	21	38%
	No	34	62%
ACLR (double‐bundle)	Yes	13	24%
	No	42	76%
ACLR (hamstrings)	Yes	20	36%
	No	35	64%
ACLR (quadriceps tendon)	Yes	1	2%
	No	54	98%
Meniscus surgery was a revision procedure	Yes	13	24%
	No	42	76%
Medial BHMT	Yes	46	84%
	No	9	16%
Lateral BHMT	Yes	11	20%
	No	44	80%
Posterior horn affected	Yes	54	100%
	No	0	0%
Pars intermedia affected	Yes	51	94%
	No	3	6%
Anterior horn affected	Yes	2	4%
	No	52	96%
Cooper zone	red‐white	2	5.7%
	red‐red	33	94.3%
BHMT dislocated into a notch	Yes	39	70.9%
	No	16	29.1%
Intraoperative bone marrow aspirate injection	Yes	30	55%
	No	25	46%
Number of sutures used		4–16	11.2 ± 3.2
Number of all‐inside		2–6	3.9 ± 1.2
Number of outside‐in		1–12	7.2 ± 2.9
Follow‐up	Months	20–158	87.5 ± 40.9

Abbreviations: ACLR, anterior cruciate ligament reconstruction; BHMT, bucket‐handle meniscal tear; SD, standard deviation.

Mean IKDC scores at the final follow‐up were 80.4 ± 17.8. Mean Lysholm scores were 86.9 ± 16.7. Mean KOOS scores were (i) symptoms: 83.6 ± 18.3, (ii) pain: 90.2 ± 14.4, (iii) activities of daily living: 93.6 ± 15.1, (iv) sports: 78.3 ± 26.0 and (v) quality of life: 70.5 ± 24.5. Mean patient satisfaction (VAS) was 7.9 ± 2.5. The mean post‐operative Tegner score was 4.9 ± 1.9. The failure rate was 9% (5 medial, 0 lateral). Further post‐operative outcome measures are detailed in Table 3.

**Table 3:** Post‐operative outcome measures. Univariate analysis of post‐operative clinical outcomes at final follow‐up.

Outcome measure	Coding/categories	Mean ± SD	Range
IKDC	0–100	80.4 ± 17.8	13.8–100.0
Lysholm score	0–100	86.9 ± 16.7	21.0–100.0
Tegner activity scale	0–10	4.9 ± 1.9	1.0–10.0
KOOS			
Symptoms	0–100	83.6 ± 18.3	14.3–100.0
Pain	0–100	90.2 ± 14.4	30.6–100.0
ADL	0–100	93.6 ± 15.1	19.1–100.0
Sports	0–100	78.3 ± 26.0	0.0–100.0
QoL	0–100	70.5 ± 24.5	12.5–100.0
Patient satisfaction (VAS)	0–10	7.9 ± 2.5	0.0–10.0
Re‐rupture	Yes	5	9%
	No	50	91%

Abbreviations: ADLs, activities of daily living; IKDC, International Knee Documentation Committee; KOOS, Knee Injury and Osteoarthritis Outcome score; QoL, quality of life; SD, standard deviation; VAS, visual analogue scale.

Patients undergoing isolated BHMT repair exhibited superior post‐operative outcomes compared to those receiving concurrent ACLR. Post‐operative IKDC scores for isolated BHMT repair were 83.6 ± 14.3, in contrast to 75.5 ± 21.7 for concurrent ACLR. However, this difference did not achieve statistical significance across most outcome measures, as detailed in Table 4.

**Table 4:** Isolated BHMT repair versus concurrent ACLR. Bivariate analysis of post‐operative outcome measures, comparing isolated BHMT repair and BHMT repair with concurrent ACLR.

Outcome measure	Coding/categories	Mean ± SD	Difference	*p* Value
**IKDC**	Isolated	83.6 ± 14.3	8.1	n.s.
	With ACLR	75.5 ± 21.7		
**Lysholm score**	Isolated	90.5 ± 11.8	9.6	n.s.
	With ACLR	80.9 ± 21.5		
**Tegner activity scale**	Isolated	5.0 ± 1.5	0.2	n.s.
	With ACLR	4.8 ± 2.4		
**KOOS**				
Symptoms	Isolated	88.8 ± 11.5	13.5	*p* = 0.024
	With ACLR	75.3 ± 23.9		
Pain	Isolated	92.6 ± 9.7	6.4	n.s.
	With ACLR	86.2 ± 19.4		
ADL	Isolated	96.2 ± 9.3	6.6	n.s.
	With ACLR	89.6 ± 21.0		
Sports	Isolated	82.6 ± 22.2	10.9	n.s.
	With ACLR	71.7 ± 30.5		
QoL	Isolated	74.8 ± 21.5	11.4	n.s.
	With ACLR	63.4 ± 27.8		
**Patient satisfaction (VAS)**	Isolated	8.0 ± 2.8	0.4	n.s.
	With ACLR	7.6 ± 2.0		

*Note*: Groups were compared using an unpaired *t* test.

Abbreviations: ACLR, anterior cruciate ligament reconstruction; ADLs, activities of daily living; BHMT, bucket‐handle meniscal tear; IKDC, International Knee Documentation Committee; KOOS, Knee Injury and Osteoarthritis Outcome Score; n.s., not significant; QoL, quality of life; SD, standard deviation; VAS, visual analogue scale.

There was a consistent positive correlation between the total number of sutures used and post‐operative outcome measures. However, only correlations with post‐operative Tegner scores (Tau B = 0.23, *p* = 0.031) and KOOS Activities of Daily Living sub‐scores (Tau B = 0.22, p = 0.035) reached statistical significance (cf. Table 5). Furthermore, medial BHMT repair achieved superior results compared to lateral repair, as detailed in Table 6, with post‐operative IKDC scores of 82.7 ± 15.9 and 69.2 ± 23.3 (*p* = 0.038), respectively. While all failures involved the medial meniscus, this association was not statistically significant (*p* = 0.3). No significant influence of age at the time of surgery (30 years or older vs. <30 years) on post‐operative outcome measures was observed.

**Table 5:** Relationship between number of sutures and clinical outcome measures. Analysis of the correlation between the number of sutures used for BHMT repair (all‐inside *and* outside‐in) and post‐operative outcome measures, using the Tau B correlation coefficient.

Outcome measure	Coding/categories	Correlation	*p* Value
IKDC	0–100	+0.125	n.s.
Lysholm score	0–100	+0.141	n.s.
Tegner activity scale	0–10	+0.228	*p* = 0.031
KOOS			
Symptoms	0–100	+0.059	n.s.
Pain	0–100	+0.125	n.s.
ADL	0–100	+0.222	*p* = 0.035
Sports	0–100	+0.180	n.s.
QoL	0–100	+0.131	n.s.
Patient satisfaction (VAS)	0–10	+0.097	n.s.

*Note*: The independent variable was the total number of sutures used.

Abbreviations: ADLs, activities of daily living; BHMT, bucket‐handle meniscal tear; IKDC, International Knee Documentation Committee; KOOS, Knee Injury and Osteoarthritis Outcome Score; n.s., not significant; QoL, quality of life; VAS, visual analogue scale.

**Table 6:** Medial versus lateral BHMT repair. Bivariate analysis of post‐operative outcome measures, comparing medial and lateral BHMT repair.

Outcome measure	Coding/categories	Mean ± SD or percentage	*p* Value
IKDC	Medial meniscus not affected	69.2 ± 23.3	*p* = 0.038
	Medial meniscus affected	82.7 ± 15.9	
Lysholm score	Medial meniscus not affected	79.7 ± 20.9	n.s.
	Medial meniscus affected	88.3 ± 15.6	
Tegner activity scale	Medial meniscus not affected	3.7 ± 1.4	*p* = 0.030
	Medial meniscus affected	5.2 ± 1.9	
KOOS			
Symptoms	Medial meniscus not affected	75.0 ± 23.4	n.s.
	Medial meniscus affected	85.3 ± 16.9	
Pain	Medial meniscus not affected	84.3 ± 20.3	n.s.
	Medial meniscus affected	91.3 ± 12.9	
ADL	Medial meniscus not affected	85.8 ± 25.3	n.s.
	Medial meniscus affected	95.2 ± 12.0	
Sports	Medial meniscus not affected	65.6 ± 31.5	n.s.
	Medial meniscus affected	80.9 ± 24.4	
QoL	Medial meniscus not affected	55.6 ± 23.5	*p* = 0.045
	Medial meniscus affected	73.4 ± 23.8	
Total	Medial meniscus not affected	79.6 ± 23.4	n.s.
	Medial meniscus affected	89.2 ± 14.3	
Patient satisfaction (VAS)	Medial meniscus not affected	5.3 ± 3.9	*p* = 0.047
	Medial meniscus affected	8.4 ± 1.8	
Re‐rupture	Medial meniscus not affected	0%	n.s.
	Medial meniscus affected	11%	

*Note*: Groups were compared using an unpaired *t* test.

Abbreviations: ADLs, activities of daily living; BHMT, bucket‐handle meniscal tear; IKDC, International Knee Documentation Committee; KOOS, Knee Injury and Osteoarthritis Outcome Score; n.s., not significant; QoL, quality of life; SD, standard deviation; VAS, visual analogue scale.

## DISCUSSION

This comparative, retrospective case series investigated clinical outcomes after arthroscopic repair of large BHMTs in 55 patients at a mid‐ to long‐term follow‐up of 7.3 years. The key findings were: (i) arthroscopic repair of large BHMTs achieved good clinical outcomes at a low failure rate of 9%, (ii) concurrent ACLR did not meaningfully impact clinical outcomes after BHMT repair, and (iii) medial BHMT repair achieved superior results compared to lateral repair.

Meniscal repair presents clear advantages over meniscectomy for BHMTs by preserving meniscal tissue and native biomechanics, preventing the accelerated degeneration associated with meniscectomy [1, 12, 19, 23, 40]. This study provides evidence supporting arthroscopic repair as an effective treatment strategy, specifically for large BHMTs. Overall, these results indicate that arthroscopic repair yields good clinical outcomes, with a comparatively low risk of failure, observing a failure rate of 9%. This aligns with recent systematic reviews reporting failure rates between 14.8 and 29.3% for BHMT repair [5, 8].

The mean post‐operative IKDC score in the present study was 80.4 ± 17.8, aligning with previous literature reporting scores between 70.0 and 92.3 [15, 16, 27, 42]. Notably, the study reporting the highest post‐operative IKDC scores, focused on patients aged 18 years or younger, prohibiting direct comparisons [15]. Furthermore, good post‐operative KOOS scores were observed in this study, akin to those reported by Goh et al. [14].

A 9% failure rate was observed at a 7‐year mean follow‐up, lower than in most previous studies, and aligning with a recent systematic review by Costa et al. reporting an estimated pooled failure rate for BHMT repair of 14.8% [8]. The failure rate in the present study was also lower than the 29.3% failure rate reported in the systematic review by Ardizzone et al. focusing on all‐inside BHMT repair techniques [5]. Another study in a limited‐resource setting reported a 10.5% clinical failure rate after BHMT repair using primarily outside‐in techniques. While failure rates in this study were similar, direct comparisons are difficult, given differences in setting and surgical technique. Nonetheless, this strongly supports arthroscopic BHMT repair as an effective treatment approach, even in a low‐resource setting [17]. Overall, these rather considerable differences in failure rates might be attributable to heterogeneity in surgical techniques, patient selection criteria, definition of ‘failure’, and preoperative joint status. The comparatively low failure rates in the present study may indicate superior repair survival when utilising a hybrid technique with a relatively high number of sutures, especially for large tears extending from the posterior horn into the pars intermedia. However, direct comparisons remain difficult, especially due to varying definitions of failure across studies.

Interestingly, while no statistically significant difference between isolated BHMT repair and concurrent ACLR was observed, patients undergoing isolated BHMT repair exhibited superior post‐operative outcomes, contradicting some previous studies hypothesising a positive influence of concurrent ACLR on meniscal repair [9, 10, 29, 37], and akin to a number of previous publications finding no significant differences between isolated meniscal repair and concurrent ACLR [3, 21, 31, 38, 42]. While preoperative joint status might influence these results, the fact that post‐operative outcomes after BHMT repair with concurrent ACLR were in fact inferior to those after isolated repair calls into question the belief that simultaneous ACLR enhances meniscal healing.

Furthermore, significantly better patient‐reported outcomes were observed after medial BHMT repair compared to lateral repair, contradicting previous reports which found no significant differences [5, 32]. However, while not statistically significant, all failures in this cohort involved medial BHMT repair, aligning with Kalifis et al. [21] and Costa et al. [8] reporting a higher risk of failure after medial meniscus repair. As such, poorer medial meniscal healing could be related to higher biomechanical stresses on the medial meniscus [24]. Surgeons may consider additional reinforcement techniques or meticulous rehabilitation for medial BHMT repairs or anticipate a higher risk of failure with medial BHMT repair.

Finally, while not reaching statistical significance for most outcome measures, possibly due to the relatively small sample size, a consistent positive correlation between the total number of sutures and post‐operative outcome measures was observed and suggests that using a larger number of meniscal sutures might be beneficial in the repair of large BHMTs. Further investigation is needed, for example, targeted biomechanical studies, to substantiate this hypothesis.

### Limitations

This study has several important limitations. The retrospective study design inherently limits conclusions about causality and increases the risk of bias, and the relatively small sample size with considerable loss to follow‐up led to several differences between groups not reaching statistical significance. Furthermore, the study population exhibited notable heterogeneity and included a significant number of revision procedures. Selection bias could have occurred, due to only including patients available for follow‐up. Attempts were made to minimise this by contacting all eligible patients treated in the defined study period. The single‐surgeon data from a specialised clinic may limit generalisability. Additional studies across multiple centres could provide more robust evidence. Furthermore, despite rigorous examinations, asymptomatic re‐tears cannot be fully excluded. Moreover, patients rated their satisfaction and outcomes at a single postsurgical timepoint, which could vary from assessments made earlier or later after surgery. Finally, while a 7‐year mean follow‐up provides valuable mid‐ to long‐term data, longer follow‐ups are still needed, to demonstrate potential osteoarthritis prevention and lifetime benefits of BHMT repair versus meniscectomy.

## CONCLUSIONS

Arthroscopic BHMT repair achieved good clinical outcomes and an acceptable failure rate of 9% at a mean follow‐up of 7 years, supporting the clinical value of meniscal repair, including large BHMTs. Concurrent ACLR showed no impact on clinical outcomes.

## AUTHOR CONTRIBUTIONS

Johannes Pawelczyk and Rainer Siebold conceptualised and designed the study. Johannes Pawelczyk carried out data collection, manuscript preparation, and manuscript revision. Ilias Fanourgiakis, Sven Feil, Ioannis Kougioumtzis and Maja Siebold were involved in drafting and revising the manuscript. Rainer Siebold led the design and conceptualisation of the study, performed the surgical procedures and critically revised the manuscript. All authors reviewed and approved the final draft of the manuscript.

## CONFLICT OF INTEREST STATEMENT

The authors declare no conflict of interest.

## ETHICS STATEMENT

ATOS Clinic Heidelberg, Ethics Committee, Bismarckstr. 10‐15, 69115 Heidelberg, Germany. Chairman: Prof. Dr. med. Christoph Becher. Reference number: 32021. Written informed consent was obtained from all patients prior to enrolment in the study.

## Data Availability

The data used during this study are available from the corresponding author upon reasonable request.
